# Reducing the impact of cardiovascular disease in older people with cancer: a qualitative study of healthcare providers

**DOI:** 10.1007/s11764-023-01331-2

**Published:** 2023-01-17

**Authors:** Reegan Knowles, Emma Kemp, Michelle Miller, Bogda Koczwara

**Affiliations:** 1 College of Medicine and Public Health, Flinders University, Adelaide, South Australia Australia; 2 College of Education, Psychology, and Social Work, Flinders University, Adelaide, South Australia Australia; 3 Caring Futures Institute, Flinders University, Adelaide, South Australia Australia; 4 College of Medicine and Public Health, Flinders Medical Centre, Flinders University, Adelaide, South Australia Australia

**Keywords:** Cardiovascular disease, Cancer, Healthcare providers, Perceived needs, Older people

## Abstract

**Purpose:**

Cancer survivors are at greater risk of cardiovascular disease (CVD) than cancer-free controls. Despite evidence-based guidelines recommending CVD risk factor assessment, surveillance and risk-reduction, many people with cancer do not receive adequate CVD care. To address potential barriers and enablers of care, we examined healthcare professionals’ (HCPs) perceptions and experiences of CVD risk assessment and management in people with cancer.

**Methods:**

We conducted one focus group and 12 individual interviews to examine HCPs’ perceptions and experiences of CVD care in care. We used reflexive thematic analysis to collect and analyse the qualitative data to construct and understand themes.

**Results:**

Twenty-one HCPs participated (8 oncologists, 5 nurses, 3 general practitioners, 2 dietitians, 1 cardiologist, 1 haematologist and 1 physiotherapist). Majority of HCPs were aware of CVD risk in cancer but were concerned they could not deliver CVD care alone due to system-level barriers including lack of time and training. HCPs also perceived patient-level barriers including socioeconomic disadvantage and fatalistic outlook. Despite barriers, HCPs suggested diverse solutions for improving CVD care in cancer including new models-of-care, clinical pathways, risk assessment/management tools and education.

**Conclusions:**

The diversity of perceived barriers and suggested solutions identified by HCPs suggests the need for a multilevel approach tailored to context. Future research involving people with cancer is needed to co-design acceptable interventions.

**Implications for Cancer Survivors:**

Improved understanding of HCP’s perceptions can inform the development of new interventions to deliver CVD care to people with cancer to reduce morbidity and mortality.

## Background

Cardiovascular disease (CVD) is a major cause of competing mortality and morbidity in people with cancer because of shared risk factors such as physical inactivity and smoking and the cardiotoxic effects of common anti-cancer treatments [1–4]. Older people with cancer, compared to their younger counterparts, are at greater risk of experiencing comorbidity [5–7] and have a higher all-cause and CVD mortality rate [8].

Evidence-based guidelines recommend CVD risk factor assessment, surveillance and risk-reduction as best practice supportive/survivorship care of people with cancer [9]. A wealth of evidence supports encouraging people with cancer to engage in healthy lifestyle behaviours, including physical activity [10–12], healthy diet [10], smoking cessation [13] and moderation of alcohol consumption [14]. However, many people with cancer do not receive appropriate assessment and monitoring/management of CVD and its risk factors [15], and older individuals with cancer are even less likely to be assessed for CVD risk than their younger counterparts, with preventive health services typically decreasing with age [16]. Understanding is limited as to why CVD risk identification and management is not routinely undertaken; however, data suggests a lack of guidance and resources to assist clinicians to identify risk [17] and lack of resources to manage this risk [18, 19]. Greater depth of understanding is needed about barriers, as well as preferences for CVD risk identification and management. Focused understanding of these issues particularly in older people with cancer is warranted given they are more likely to experience comorbid CVD, have higher all-cause mortality and may have different perceptions regarding barriers to and preferences for reducing the impact of CVD in cancer.

To address this gap, this study aimed to examine health care professional’s (HCP) perceptions and experiences of the management of CVD and risk in older people with cancer.

## Methods

### Design

This qualitative study was undertaken as part of the broader program of research which aims to develop a new approach to CVD risk factor management in older people with cancer. The study involved focus groups and interviews conducted in person, via telephone and online.

### Recruitment

HCPs were recruited through researchers’ existing networks using a non-random, purposive sampling technique. Potential participants were informed of the project aims and study procedures (verbally and via the Participant Information and Consent Form [PICF]) before they indicated consent by signing the PICF. Participants could choose whether they participated via focus group or individual interview, and in-person, online or telephone.

### Study procedures

Data was collected via focus group or individual interview. All interviews and focus groups were conducted by the same investigator (RK) with assistance from another (EK) for the focus group. The sessions were semi-structured using a topic guide (see Appendix 1) to collect the perceptions and experiences of HCPs around the central phenomenon of CVD in older people with cancer. Recruitment continued until the researchers perceived enough data to conduct rich and meaningful analysis and to develop a report that is interesting, informative and can inform future research and clinical practice. This approach aligns with the premise that “new” data can always be collected and ceasing data collection is a pragmatic decision based on the objectives of the research [20].

### Data analysis

The focus group and interviews were audio-recorded and transcribed verbatim. We used the RTA approach to data analysis, underpinned by a critical realist paradigm to address our research objectives. RTA involves the development, analysis and interpretation of a range of qualitative data in which the researcher is involved in the construction of knowledge around a central phenomenon [21]. We used NVivo to assist with coding and theme development, and our analysis progressed through frequent discussions involving all researchers.

The analysis was conducted according to the six phases of the RTA framework [21] (i.e. data familiarisation, coding, initial theme development, review of themes, refinement, reporting). Semantic and inductive analysis predominated our approach to the generation of themes, in which the researchers identified themes explicitly communicated by the participants during focus groups and interviews. However, to increase the depth and richness of analysis, we also employed a latent approach to the analysis of some themes, in which we further discussed and analysed data to uncover the underlying meaning of participants’ contributions. We practiced reflexivity throughout the analysis process to identify, understand and consider the impact of our own perspectives, perceptions and beliefs on the construction of knowledge in our research. The data analysis approach was iterative and recursive, as discussions and drafting of findings often led to new ideas and perspectives which required further discussion and analysis. An example of the flexible and iterative approach we used during both data collection and analysis was that although we aimed to encourage discussion about CVD and cancer with a specific focus on older people, we identified that many responses did not include differentiation between older and younger people. This was unpredicted but we determined that it occurred organically through the research process and chose to allow flexibility and authenticity in the application of the research methodology.

### Ethics

Ethics approval was granted by the Southern Adelaide Local Health Network Ethics Committee on 22 January 2020 (HREC/19/SAC/309).

## Results

A total of 21 HCPs participated in the research. Nine HCPs participated in one online focus group which lasted 59 min; and 12 HCPs participated in an individual interview (face-to-face *n* = 9; telephone *n* = 2; online *n* = 1), ranging from 12 to 53 min duration (median = 36 min).

**Fig. 1:**
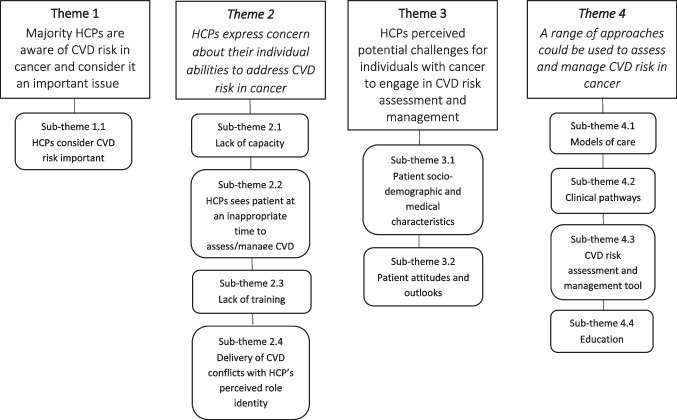
Themes and sub-themes summarising HCP’s perceptions of CVD risk identification and management in older people with cancer

The analysis identified four themes and 11 subthemes (Fig. 1).

### Theme 1: Majority of HCPs are aware of CVD risk in cancer and consider it an important issue to address

Majority HCPs indicated that they were aware people with cancer had a higher CVD risk and summarised reasons for the co-existence of these diseases. For example:

…toxicity of treatments we do for cancer, so for many of our chemotherapy or target treatments have a cardiovascular effect – that’s one. Second is that some cancers have similar risk factors for development of the cancer as to cardiovascular disease, so its obesity, diet, smoking* –* Medical oncologist

But one HCP (GP) mentioned they were not aware of the impact of cancer diagnosis on CVD risk.

I guess I’m not really well aware of that impact of increased risk [of CVD with history of cancer)…I don’t think in my mind I factor in specifically as cancer survivors being at higher risk unless specifically their treatment has been flagged as being a potential risk factor. *–* GP

#### Sub-theme 1.1: HCPs consider CVD risk important

HCPs from a range of disciplines highlighted perceived importance of CVD risk in cancer. There was a strong focus on the perceived importance of weighing up the (cardiotoxic) risk and (anti-cancer) benefit of anti-cancer treatment.

And if a patient has, for example, say, 20%, 30% risk of death just because of their cardiovascular risk it doesn’t make sense to give them toxic treatments for 1% or 2% improvement. – Medical oncologist

Intervention to reduce CVD risk was identified as potentially improving patients’ quality of life.

…they’ve [typical lung cancer patient getting high dose radiotherapy] got a 20% chance of having a CV event in 2 years. Yet those patients are often not referred because you got people saying they’ve got such a poor prognosis there’s no point. But if you could potentially prevent a cardiovascular event from a QOL perspective in the few years they’ve got remaining, why wouldn’t you…? – Cardiologist

### Theme 2: HCPs expressed concern about their individual abilities to address CVD risk in cancer

Majority of participants expressed concerns about their ability to deliver CVD care to people with cancer because of lack of capacity, training, inappropriate timing or perception of CVD care as outside of their role identity.

#### Sub-theme 2.1: Lack of capacity

Lack of time was the most common barrier to delivery of CVD care. For example, dietitians discussed how time limitations precipitated the need for prioritisation of cancer-related needs over CVD, and the need for referral of patients with CVD-related issues to community dietetics services.

I think it’s all about priorities and so because everyone has limited time everyone aims at looking at the cancer. – Dietitian

A lack of adequate resources and services and inadequate cohesion/coordination of tasks by different professions involved in cancer care were identified as potential barriers to the provision of CVD care in cancer. A cardiologist commented that there was an inadequate number of cardiologists appropriately trained for providing cardio-oncology care and linked this to the absence of training programs.

there’s probably only about 30 cardiologists in the country that have got an interest, there’s 2 that are certified with the ICS which is sort of the de facto forming international group interested, and there’s about 2 or maybe 3 who have done international overseas fellowships. There’s no training programs in Australia, no requirement for training, so we couldn’t deal with all the work anyway. – Cardiologist

A dietitian mentioned that hospital-based dietitians did not have the capacity to address CVD care in people with cancer, as their focus is on dealing with acute problems directly related to cancer.

we’re just trying to make sure that we meet nutritional requirements, there’s a lot of issues with inability to eat so it’s not often around food groups or what you focus on when you think about cardiovascular health, because we’re just trying to help keep them alive really and then the patients we keep long-term it’s often around trying to improve quality of life. So, our work is very acute. – Dietitian

Participants communicated their perception that many patients did not receive survivorship care/post-treatment care, with one participant suggesting that this would be where CVD risk management would best fit.

…if the person comes out of the hospital with a clear survivorship plan, then there’s a fair chance that it will be followed, but most people don’t come out with a survivorship plan. – GP

A cardiologist identified that oncology and cardiology professions did not work together cohesively or communicate effectively, and this lack of connection between disciplines was perceived as a barrier to CVD care delivery.

…there is a disconnect between oncology and cardiology because there are different journals,… different language,…different side effects, reporting algorithms, different conferences and we sit on two sides of a chasm. It’s only in the more recent past that there’s been more movement towards bringing those two sides together – Cardiologist

#### Sub-theme 2.2: The time HCPs consult with patients is not the right time to raise CVD risk

Some participants expressed concern about *timing*; i.e. some felt the time at which they see their patient was not appropriate to introduce more information about another potential health problem. For example,

…when you see somebody for the first time you have to talk to them for nearly an hour about what their disease means and the side effects of the treatment and all of that and that’s without at all talking about vascular risk status and so, I guess, partly that’s an issue for my time because it already runs over but it’s also an issue for them because they’re often frightened and overwhelmed and it’s not really the time to be trying to pile on more information. – Haematologist

…people have to be ready to take information on board, I don’t think during the peak time of treatment would be so cool [to provide CVD risk assessment/education] –Dietitian

#### Sub-theme 2.3: Lack of training

HCPs (including a GP, oncologist and cardiologist) perceived inadequate training/education to be a barrier to the delivery of CVD care in cancer.

I don’t feel like I have any guide, we know for example managing high BP, if you’re a diabetic, your targets are much lower. If you’ve got a preexisting cancer, so am I aiming for a better target? What are the more important things to manage in terms of decreasing their CV risk? I don’t think it’s beyond our scope, I just feel like we don’t have the tools and the guidelines to direct those interventions precisely. – GP

#### Sub-theme 2.4: Delivery of CVD conflicts with HCP’s perceived role identity

Many HCPs communicated their perception that delivering CVD care did not align with how they saw their professional role. Some HCPs explicitly described that they (or their discipline) were not appropriate to deliver CVD care. For example:

I’m not up to date on the optimum level to diabetes or blood pressure and I’m not interested in acquiring that knowledge. – Haematologist

I don’t see my role managing patients’ cardiovascular health. I think what I can do is assess how much I’m going to cause damage and whether or not that treatment is worth doing. But whether or not they need their hypertension to be controlled better, the most I can do is write a letter to the GP. – Medical oncologist

HCPs communicated that they perceived other professions as more appropriate for providing CVD assessment and management. For example,

whether the head clinician likes it or not, they’re still the gatekeeper for a lot of referrals [as part of CVD care] – Dietitian

[patients could receive CVD care] if you had a nurse practitioner leading that program and the other nurse practitioners knew that… – Dietitian

Nurses identified GPs as being the most suitable for addressing CVD risk in patients because.

It’s part of coordination and care and that’s the GP, not like a snapshot of a nurse or a dietitian who has like one episode. – Nurse

A GP suggested that patients could play a role in the CVD care process through consultation with their healthcare team.

Some of them [patients] I think are so motivated and very invested in their health… GP

A haematologist stated that any of multiple professions could achieve this role.

I wouldn’t say that there’s one person to initiate that conversation, I can see lots of people being good people to initiate that conversation and that ranges from the GP, the medical oncologist, the surgeon, of the surgeons referring for radiation therapy, you know, the rad oncs, other people that are involved, breast care nurses, you know the McGrath foundation nurses, anybody that is involved in the care is in a good position to highlight the thought in the patient that they should think about it. – Haematologist.

### Theme 3: HCPs perceived potential challenges for individuals with cancer to engage in CVD risk assessment and management

HCPs identified individual characteristics of patients with cancer that they perceived as barriers to engaging in CVD care. Some of these were demographic or medical factors, including level of disadvantage and prognosis. Other characteristics were related to patient attitudes and outlooks, such as low motivation and a fatalistic outlook.

#### Sub-theme 3.1: Sociodemographic and medical characteristics

One participant indicated that a patient’s engagement in CVD assessment and management would be impacted by aspects associated with a patient’s socioeconomic situation, e.g. financial barriers, health literacy and access to healthcare.

it’s so difficult to predict who’s going to react [to CVD assessment/management] because it’s just all about the person and their socio-economic situation… – GP

Being isolated was also identified as reducing the likelihood that some patients would engage in CVD assessment/management.

Always having to reach the people [to provide them with CVD care] that have actually distanced themselves through it all [cancer treatment], they’re the harder – the unreachables, so that’s always the challenge. – GP

#### Sub-theme 3.2: Patient attitudes and outlooks

One nurse and one GP discussed their perceptions that aspects of a patient’s outlook or mindset would reduce their engagement in CVD care, including: being in denial about their disease, having an “alternative” approach to healthcare, being unwilling/reluctant to make changes, being less demanding/entitled and having a fatalistic outlook.

But it’s also denial. All these people with their inbuilt defence mechanisms, so that’s [introducing CVD care] really, really difficult. – GP

I’ve had one or two [patients] who are very alternative so I guess the prospect of … starting any other medication is … revolting [to the patient]. – Nurse.

one or two [patients] are like “I’m not quitting smoking, it’s my only joy in life if I die of it then I’m ok.” – Nurse.

Being less demanding and having a fatalistic outlook were both specifically raised in the context of older people with cancer:

…they [the older person with cancer] almost have a sense of being a bit fatalistic, like I’m getting older so why would I bother? – GP

…they [the older person with cancer] come into the doctor and they don’t want to waste your time [asking questions about CVD risk], they’re a bit more old school, they’re not as entitled or as demanding… – GP

Other participants noted that they perceived patients may interpret the offer of CVD care as overservicing or exploitation:

…some of the things they [the patient] are worried about is that this is just a money-making thing for the business and that’s why you’re referring, that goes down very poorly. – Cardiologist

### Theme 4: A range of approaches could be used to assess and manage CVD risk in cancer

HCPs discussed a diverse range of solutions and approaches which could improve delivery of CVD care in cancer, including new models of care, clinical pathways, tools and education. In addition, participants discussed the specific components they perceived as important in approaches to CVD care in cancer, including automaticity, communication, and a patient-centred approach.

#### Sub-theme 4.1: Models of care

HCPs discussed a range of models of care for the identification and management of CVD in individuals with cancer. These included cardio-oncology clinics; care models led/coordinated by selected professions (i.e. GPs and nurses); and multi-disciplinary/teams-based models.

A range of HCPs from nursing, haematology and allied health discussed the potential for a cardio-oncology clinic. For example:

I would prefer it if there were a kind of cardio-oncology clinic that could see people, even if it’s once, and make an assessment and give them a package of advice. – Haematologist

HCPs (including a nurse) suggested a nurse-led model of care, such as that used in other areas of care, could also deliver CVD care:

I think that if you look at it in cardiology, cardiac rehab has these nurse-led multi-d team…, there’s heart failure, there’s hypertension and there’s AF [atrial fibrillation], this is just an extension of those models of care, in reality we’re not reinventing the wheel, we’re just stealing basically. – Nurse

Nurses highlighted the role of the GP in coordination of care, implying that this role makes GPs most appropriate for coordinating a cardio-oncology model of care.

GPs coordinate care. A lot of our referrals more than often are from GPs…that’s where we get medical histories from…the GP is getting the letters from us, from oncology, …. the physio, …. lymphedema assessment from the radiotherapist… So, they’re the linchpin. – Nurse

Multi-disciplinary or teams-based approaches to care were discussed by a range of HCPs. Several asserted the success of multi-disciplinary teams in other areas of care, and highlighted that by involving a range of professions in the model of care less responsibility is felt by individuals.

Well, I guess, I think the ideal arrangement [is] … a multidisciplinary team with cardiologists and specialists and so on together with the dietician and educator what have you. – Haematologist

…you kind of build up a bit of a team and sort of a collaboration so people don’t feel like they’re always bothering the same person… – Physiotherapist

#### Sub-theme 4.2: Clinical pathways

HCPs discussed the potential for a clinical pathway to ensure coordinated CVD care in cancer.

“Well, ideally there would be a referral pathway.” – Dietitian

“Yeah. I think it should be referral and integration so that the messages are transferred over to GP practice as well so when they move to specialty care that you have a clinical pathway where you’ve got integrative care.” – Nurse

“I think there needs to be a systematisation of the workflows, and that has to be balanced up against the workforce capacity to deliver the care.” – Cardiologist

HCPs asserted the importance of protocols in providing care.

“that’s how these things will work best, you have a protocol, a system that just gets followed based on the best available evidence.” – Cardiologist

However, there was also discussion about how protocols are specific to the health service and are unlikely to be transferrable to other services.

the trouble [with developing a CVD risk reduction approach/intervention] is that every location, geographically, will have a slightly different solution in terms of the process because of the way pre-existing processes are there. – Cardiologist

#### Sub-theme 4.3: CVD risk assessment and management tool

Several participants voiced their support for the development and integration of CVD risk assessment and management tools to improve CVD care in cancer, including treatment decision-aides, risk stratification and education tools.

Participants varied in their opinions regarding what would be the most useful purpose of a tool in the area of cardio-oncology, with some asserting the need for the tool to aid in treatment decisions whilst others indicated they would prefer the tool to stratify patients according to level of risk so that care provision can be triaged accordingly.

a tool… that could provide me with some evidence in terms of that person’s cardiovascular risk and particularly if it told me that they had a high risk of dying because of their heart problems, then that might actually influence the decision for the [anti-cancer] treatments that might be recommended for that patient – Nurse practitioner

Most HCPs preferred a digital tool, but some mentioned barriers to this indicating that some clinicians may be more likely to use a pen and paper version. Ipads, websites, and smartphones were identified as potential delivery modes for a digital tool.

[the tool has to be in the form of] either a phone app or it has to be online – Nurse

I think probably online portal, like I could just have it as a bookmark and then when I was seeing patients just kind of flick to that. – Physiotherapist

HCPs from a range of professions asserted the importance of specific aspects/components of a tool to deliver CVD care. Participants identified automaticity and embedding the tool into a current system (e.g. limited need for HCP to be involved in data collection/analysis) as being crucial.

…I think if you could use something … that’s running in the back of EMR [electronic medical records] and pulls all the information and spits you out a risk, I think that would be helpful because then you know who to target. – Dietitian

I don’t [want to] have to somehow input it [data about CVD assessment and management] back into the notes – GP

A lot of these kinds of tools, they should be automated into our work – Medical oncologist

Participants indicated that a tool should also facilitate communication, particularly between HCPs involved in the patient’s care.

I just think there needs to be some sort of feedback to them [other HCPs], if you’re doing some sort of screening that this has happened. - Medical oncologist

Participants identified that not all patients would be appropriate for the administration of a tool, and tools should be flexible so they can be tailored to the individual needs of different patients.

What I’m trying to actually say is that these tools are helpful, but we cannot actually use that on every patient, it just needs to be identify the situation where we can actually use it or individualise it for patients. – Medical oncologist

#### Sub-theme 4.4: Education

Participants also identified education and awareness in both HCPs and patients as important in delivering CVD care to people with cancer. A GP communicated that they would feel more capable of delivering CVD care if they were educated appropriately.

It’s some guidelines or education [of GPs] that’s kinda missing. – GP

Education of patients was identified by several participants as having a positive impact of integrating CVD care into cancer. This could, for example, lead to patients initiating conversations with HCPs about their CVD risk profile, and being more involved in the management of their own risk.

I think it’s education of providers, I think its education of patients, particularly where we’re looking at patient-centric care, patients being more involved in their health care making decisions, I think patients need to be made more aware. – Cardiologist

## Discussion

The present study presents a comprehensive qualitative analysis of the perceptions of HCPs regarding the identification and management of CVD in older people with cancer. We found HCPs were aware of CVD risk in cancer and perceived it to be important but had concerns about their own ability to deliver CVD care. We also found a lack of consensus of HCPs’ perceptions regarding the best way to reduce the impact of CVD in cancer.

Previous research specifically examining HCPs’ perceptions regarding cardiotoxicity of anti-cancer treatment reported some HCPs were not aware of the increased risk and associated poor outcomes of CVD in cancer patients and those that were aware did not perceive the relationship between CVD and cancer to be an important issue. For example, Koop and colleagues [22] reported that majority participants (Dutch oncologists, *n* = 12) were unaware of the incidence of cardiotoxicity and perceived cardiac surveillance as burdensome, and a recent survey (*n* = 190 Dutch cancer and cardiac specialists) found only 33.2% were concerned about the cardiotoxic effects of anti-cancer treatment [23]. However, in line with our research, a survey of 106 cardiology specialists in the US found that > 70% perceived potential CVD complications as an important consideration of anti-cancer treatment [24]. Many factors may influence awareness and perceived of CVD risk in cancer. For example, training and education in CVD risk in cancer vary across institutions [24]; and individual HCPs may have different preferences/interest in cardio-oncology [25]. It is possible that our findings reflect a growing awareness of the field of cardio-oncology through recent publications of recommendations and guidelines in this area [26].

A novel finding of our research was that HCPs were concerned about their (and their profession’s) abilities to deliver CVD care in cancer alone. This finding has important implications for the development of future approaches to improve CVD care in cancer, where CVD care, like the rest of cancer care, is likely to require a teams-based approach. In addition, we are not aware of other research highlighting a perceived conflict between delivering CVD care and role identity in HCPs involved in cancer care. Although conflicting role identity was identified as a barrier in a systematic review of 43 papers examining staff-reported barriers to the implementation of hospital-based interventions such as the administration of screening tools and behaviour-change interventions, this did not include any research involving CVD care after cancer [27]. Our finding can inform future approaches that aim to improve CVD care in cancer, e.g. suggests the importance of clarifying HCP’s expectations around their role and responsibilities, and providing them with the training they need to conduct expected tasks effectively.

Our research also identified HCPs’ perceptions regarding patient-level barriers reducing engagement in CVD care, including socioeconomic disadvantage, lack of motivation, having a fatalistic outlook and adversity to more intervention. We could only locate one previous study that reported HCPs’ perceptions regarding potential patient-level barriers [22], and this study also identified patient socio-economic disadvantage and having a fatalistic outlook as potential barriers to CVD care engagement [22]. In addition, we found that responses did not differentiate between older and younger people with cancer, which may suggest that there are no differences in perception of barriers (and preferences) related to age. Although our findings go some way to bridging the gap in evidence in this area, the critical next step of our broader research program is to examine patient-level barriers from research involving patients themselves and this will be the next step for our group. We are unaware of any previous research which has reported cancer patients’ perspectives regarding barriers to engagement in CVD care.

We identified that there is a lack of consensus for a single approach to reduce the impact of CVD in cancer, with multiple suggestions including a new model of care, a clinical pathway, education and a tool to detect and manage CVD risk. Based on the findings of this research, we propose that effectively reducing the impact of CVD in cancer requires the development and implementation of a suite of intervention types. The diversity of solutions which have emerged in our research supports a complex multi-component intervention may be needed to reduce the impact of CVD in cancer. We also propose the diversity of barriers identified in our research supports the need for a multi-pronged approach, and that any intervention should be tailored to the context in which it will be delivered and input from people with cancer.

### Strengths and limitations

The major strength of this research is the comprehensive, reflexive and flexible approach employing the RTA methodology to increase understanding of HCPs’ perceptions regarding CVD in cancer. There are also notable limitations of our research. Majority of participants were employees of one hospital and although several professions participated in our research, there were small numbers from some disciplines (e.g. one cardiologist).

## Conclusion

This qualitative study involving a range of HCPs suggests that while majority are aware of and appreciate the importance of CVD risk in cancer, they experience barriers to delivering care and suggest a diverse range of strategies for improving CVD risk identification and management. Future research involving cancer survivors is needed to co-design acceptable interventions to improve CVD identification and management in cancer.
